# Anomalous circumflex artery encircling the aortic annulus: implications for mitral valve repair

**DOI:** 10.1186/s13019-024-02779-8

**Published:** 2024-05-07

**Authors:** Michele Portoghese, Simone Mureddu, Andrea Balata, Cristina Contini, Giangiacomo Carta

**Affiliations:** 1 Departement Of Cardiothoracic And Vascular Surgery, Cardiac Surgery Unit, A.O.U. Sassari, Sassari, Italy; 2 Departement Of Cardiothoracic And Vascular Surgery, Cardiac Anesthesiology Unit, A.O.U. Sassari, Sassari, Italy

**Keywords:** Mitral valve surgery, Intraoperative complication, Coronary injury, Anomalous circumflex coronary course

## Abstract

Injury to coronary arteries during mitral surgery is a rare but life-threatening procedural complication, an anomalous origin and course of the left circumflex artery (LCx) increase this risk. Recognizing the anomaly by the characteristic angiographic pattern and identifying its relationship with the surrounding anatomical structure using imaging techniques, mainly transesophageal echocardiography (TOE) or coronary computed tomography angiography (CCTA), is of crucial importance in setting up the best surgical strategy. We report a case of anomalous origin of a circumflex artery (LCx) from the proximal portion of the right coronary artery (RCA) with a pathway running retroaortically through the mitro-aortic space. An integrated diagnostic approach using a multidisciplinary team with a cardiologist and an imaging radiologist allowed us to decide the surgical strategy. We successfully performed a mitral valvular repair using a minimally invasive minithoracotomic approach and implanting a complete semirigid ring.

## Background

An abnormal LCx vessels origin, steaming from the right coronary artery and following a retroaortic course, is a very uncommon anomaly (0.15% incidence) [1] that, if undiagnosed, considerably increases the risk of coronary damage with subsequent life-threatening complications during valve surgery [2]. Therefore, it is crucially important to recognize such a anomaly during the preoperative screening. We report our diagnostic and therapeutic strategy in a case of a patient candidated to mitral valve repair showing an unusual preoperative coronary angiogram which alerted us to the presence of an anomalous coronary branch encircling the aortic annulus.

## Case presentation

**Fig. 1:**
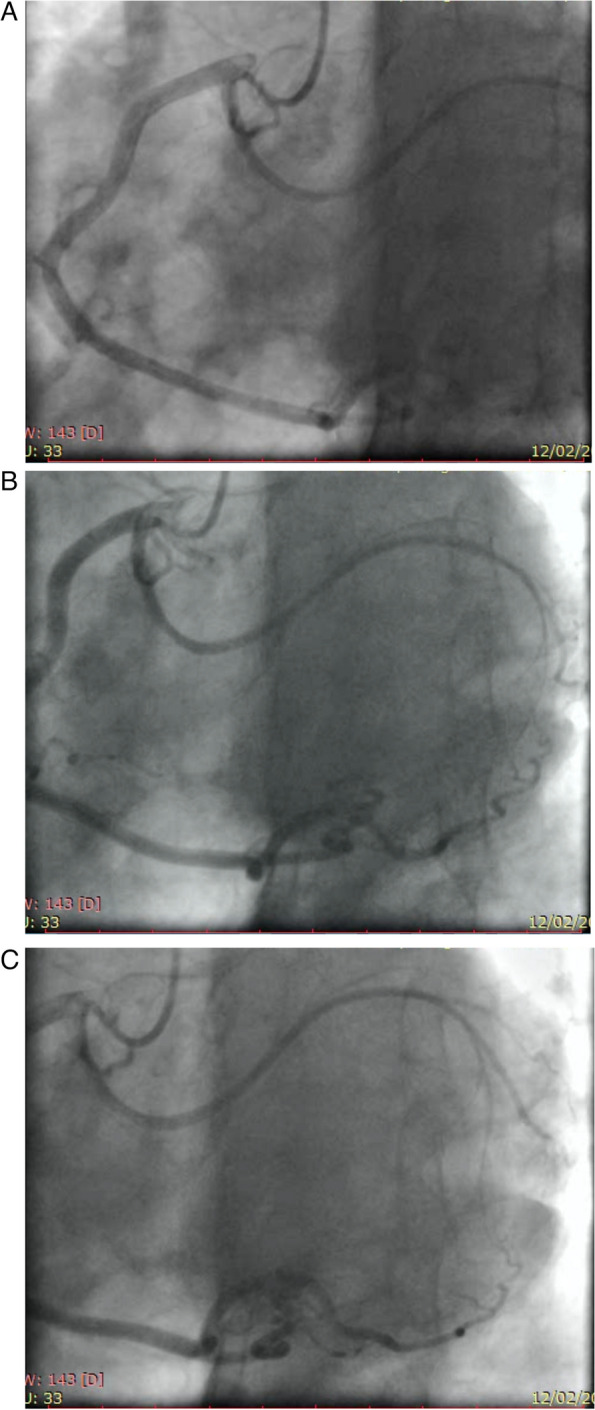
Coronary angiogram (LAO with Cranial 30°) shows the origin (1A), the middle (2A) and the distal portion (3^a^) of the anomalous circumflex artery

A 50 year old man was referred to our attention with the diagnosis of symptomatic (NYHA II/III) primitive severe mitral insufficiency. In his medical history there was no other significant events. The ECG showed sinus rhythm. Preoperative TOE echocardiography reported a huge mitral insufficiency (grade 4/4) without excess of tissue (fibroelastic degeneration), a prolapse of the anterior leaflet due to primary chordae elongation and a flail prolapse of the middle portion of the posterior leaflet (P2) due to primary chordae rupture, the annulus was dilated with a spherical shape. Indication for surgical mitral valve repair was given and preoperative routine exams were performed. The coronary angiogram showed an anomalous branch of the right coronary artery detaching earlier from the main stem and following an anomalous pathway upwards and posteriorly in the fibrous heart body along the mitro-aortic continuity (Fig. 1A, B, C).

**Fig. 2:**
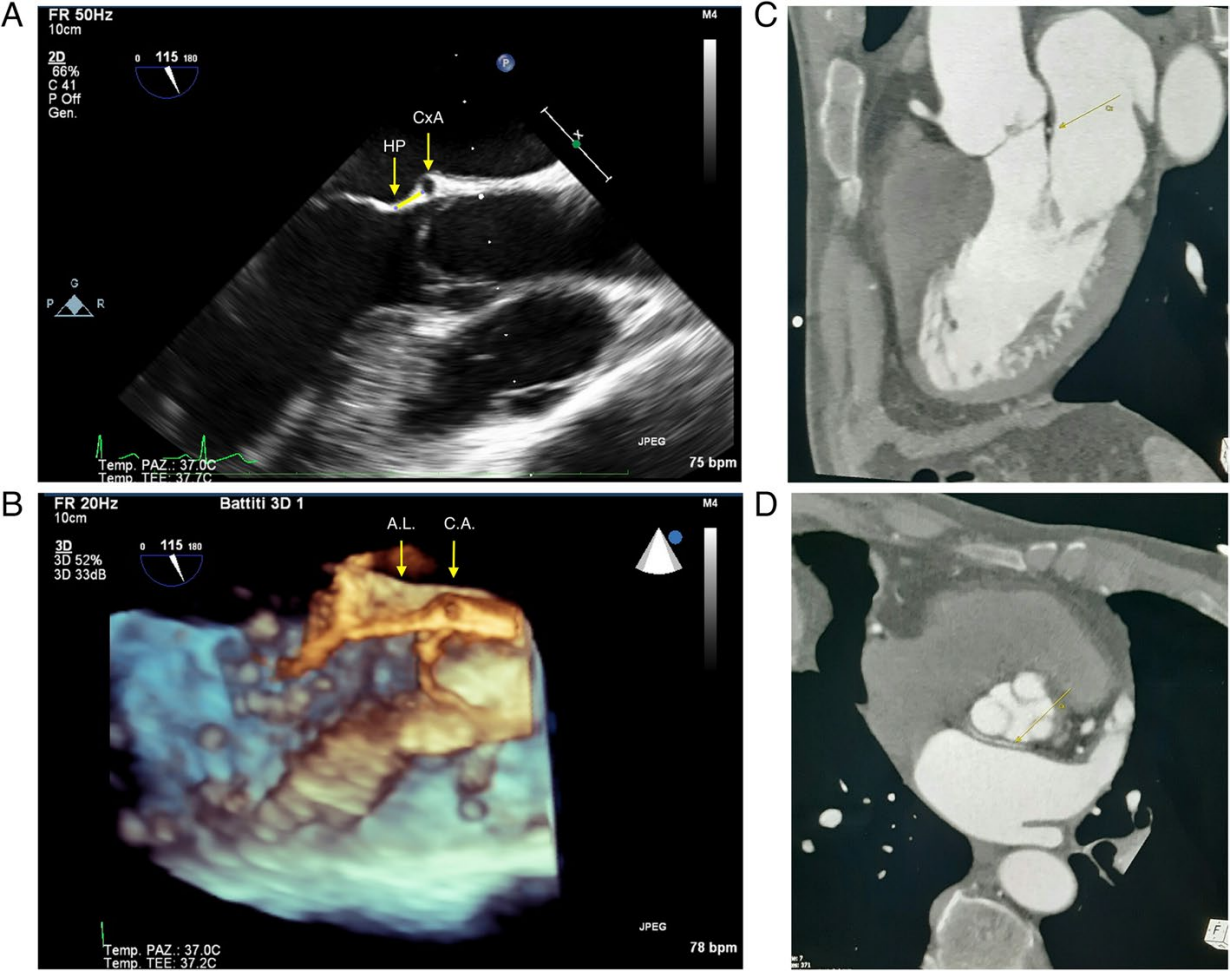
TOE long axis shows the hinge point of the anterior mitral leaflet (H.P.) and the anomalous circumflex artery (C.A.) (2A) and the distance between this two points. 2B shows the same projection in 3D. CxA (anomalous circumflex artery); HP (hinge point of the anterior mitral leaflet); AL (Anterior leaflet). IMG 2 C- 2 D The CCTA study confirmed the anomalous origin of a circumflex artery (LCx) from the proximal portion of the right coronary artery (RCA) with a pathway running retroaortically through the mitro-aortic space

The bi-dimensional (2D) TOE (Fig. 2A) confirmed the presence of the anomalous artery and the three-dimensional (3D) views (Fig. 2B) were instrumental in exactly identifying the course of the vessel which lies 65 mm upwards to the level of the hinge point between the anterior leaflet and the mitral valve annulus.

The CCTA study further confirmed the anomalous position of the vessel and its relations with the adjacent anatomical structure (Fig. 2C, D).

The operation was performed using a minimally invasive video assisted approach: right minithoracotomy (fourth intercostal space) without mechanical spreading (soft tissue retractor, Edwards Lifescience, Irvine, CA), internal aortic cross clamp (Endo-Clamp catheter, Edwards Lifescience, Irvine, CA) and cold cardioplegic arrest (Custodiol). The repair was realized according to the Carpentier technique: a triangular resection of P2, a double secondary chordae transposition on P1 and P2, implantation of two artificial chordae (GoreTeX) from the medial head of the anterior and posterior papillary muscles to A1 and A3, and a valvuloplasty with a semirigid complete ring (Physio II Edwads). Particular care was taken to precisely stitch on the anterior mitral annulus (A1, 2 and 3) exactly at the level of the mitro-annular hinge point. The immediate and post operative course went smoothly without any ECG modification or hemodynamic impairment. The patient was discharged at day 5. After 3 years of follow-up the patient is going perfectly well with normal valvular end ventricular function.

## Discussion

**Fig. 3:**
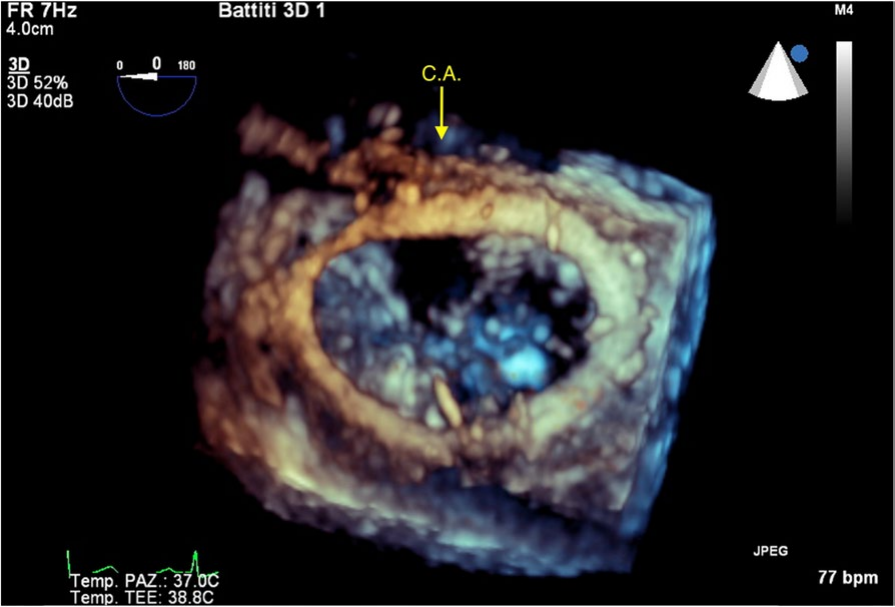
3D TOE shows the postoperative 3D view

Reparative surgery of the mitral valve for primitive mitral insufficiency is nowadays the gold standard therapy even for asymptomatic very low surgical risk patients [3]. In the last few years the use of minimally invasive techniques has been more commonly requested by patients and evidence of its feasibility and safety are increasingly reported [4]. In this setting it is crucially important to prevent any potential iatrogenic complication. The abnormal origin and retroaortic course of the LCx has a such a distinctive pattern that must act as a red flag for surgeons (Fig. 1A, B, C). That’s why, in our opinion, a preoperative coronary imaging screening for all patients candidate for mitral surgery is mandatory. Mitral valve repair with a complete ring is reported to be the treatment of choice providing a better long term durability [3, 4]. Conversely, the use of an open ring has been proposed as a safer in this condition [5], but in our opinion a correct effective implant of an open ring implies its attachment to both right and left fibrous bodies. Consequently, considered the particular course of the anomalous coronary (Fig. 2A, B), the risk of injury is not really avoided. In our opinion the most important factor to address in order to predict the safety and feasibility of a complete annuloplasty is the distance between the LCx (Left circumflex artery) and the anterior plan of the ring attachment which was about 65 mm in our case (Fig. 2A, B). The plan of the ring attachment was defined as the hinge point of the anterior leaflet which was about two millimeters more proximal to the usual stitching level (the so called “mitral annulus”). The use of the TEE echocardiography and particularly of the 3D view was crucially important in identifying the vessel before the operation and to verify its patency at the end of the procedure (Fig. 3).

## Conclusion

We wish to emphasize the vital importance of recognizing the presence of an anomalous LCx from the preoperative coronary angiogram: the image we present in Fig. 1 should be noted by every surgeon involved in valvular surgery. We strongly suggest using a multidisciplinary integrated approach to establish the best surgical technique for every specific patient.

## Data Availability

The data used to support the findings of this case report are restricted to protect patient privacy.
